# Influence of Expanded Polystyrene on the Mechanical and Tribological Performance of Alkali-Activated Lignocellulosic Ash Composites

**DOI:** 10.3390/ma19163364

**Published:** 2026-08-07

**Authors:** Gerardo Gallegos-León, Nelly Flores-Ramirez, Salomon R. Vasquez-Garcia, Leandro García-González, José J. Contreras-Navarrete, Lada Domratcheva-Lvova, Julián Hernández-Torres, José L. Rivera

**Affiliations:** 1 Department of Wood Engineering and Technology, Universidad Michoacana de San Nicolás de Hidalgo (UMSNH), Morelia 58030, Michoacan, Mexico; 1650863d@umich.mx (G.G.-L.); lvova@umich.mx (L.D.-L.); 2 Department of Chemical Engineering, Universidad Michoacana de San Nicolás de Hidalgo (UMSNH), Morelia 58030, Michoacan, Mexico; 3 Research Center for Micro and Nanotechnology, Universidad Veracruzana, Boca del Rio 94294, Veracruz, Mexico; leagarcia@uv.mx (L.G.-G.); julihernandez@uv.mx (J.H.-T.); 4 Instituto Tecnológico de Morelia, Tecnológico Nacional de Mexico, Morelia 58120, Michoacan, Mexico; jose.cn2@morelia.tecnm.mx; 5 Department of Physico-Mathematical Sciences, Universidad Michoacana de San Nicolás de Hidalgo (UMSNH), Morelia 58030, Michoacan, Mexico; jlrivera@umich.mx

**Keywords:** alkali-activated composites, wood ash, sugarcane bagasse ash, expanded polystyrene, tribological performance

## Abstract

**Highlights:**

- Lignocellulosic ashes and recycled polystyrene were valorized into lightweight composites.
- Volcanic ash outperformed metakaolin in mechanical and tribological performance.
- The hybrid ash composite achieved 7.55 MPa strength and a 0.09 mm^3^/N·m wear rate.
- Expanded polystyrene increased surface hardness but reduced compressive strength.
- Precursor composition governed the mechanical and surface performance of the composites.

**Abstract:**

Lignocellulosic waste materials (LWM), such as wood ash (WA) and sugarcane bagasse ash (SCBA), have emerged as promising alkali-activated precursors. In this study, WA- and SCBA-based matrices were modified with metakaolin (MK) and volcanic ash (VA) as complementary reactive precursors, while recycled expanded polystyrene (EPS) was incorporated as a lightweight modifier. Composites were prepared using a 15 M NaOH activator and characterized by compressive strength, Vickers hardness, and ball-on-disc tribological tests under dry sliding conditions to establish the relationship among precursor composition, mechanical behavior, and surface performance. SCBA-based composites exhibited higher compressive strength than WA-based systems, reaching 4.47 MPa in SCBA–VA formulations, whereas the hybrid WA–SCBA–VA formulation achieved the highest compressive strength (7.55 ± 0.25 MPa, *n* = 5). Although EPS reduced compressive strength, it increased surface hardness, with the highest value recorded for formulation 9BPS (25.4 HV). The WA–SCBA–VA formulation also exhibited the lowest wear rate (0.09 mm^3^/N·m), indicating superior tribological performance. Overall, VA was more effective than MK in enhancing the combined mechanical and tribological behavior of WA–SCBA-based composites. This study provides a systematic comparative assessment of the tribological performance of lignocellulosic ash-based alkali-activated composites, offering new insights into the relationship between precursor composition and surface performance in lightweight sustainable materials.

## 1. Introduction

The escalating environmental concerns associated with industrial waste accumulation and the high carbon footprint of conventional Portland cement have intensified the search for sustainable construction materials. In this context, alkali-activated materials (AAMs) have emerged as a promising class of alternative binders produced through the chemical activation of aluminosilicate-rich precursors using alkaline solutions. This process involves the dissolution of silica (SiO_2_) and alumina (Al_2_O_3_), followed by polycondensation reactions that generate amorphous or semi-crystalline binding products. Depending on precursor composition and activator chemistry, several reaction products have been reported in the literature for alkali-activated systems [1,2,3,4,5,6,7,8,9,10].

Among the available industrial byproducts, lignocellulosic waste materials (LWMs), such as wood ash (WA) and sugarcane bagasse ash (SCBA), represent an abundant and underutilized resource. With global wood extraction exceeding 4 billion m^3^ and sugarcane production reaching approximately 1.9 billion tons annually, large volumes of these residues are generated [11,12,13,14]. Due to their SiO_2_, CaO, and Al_2_O_3_ contents, these ashes have potential for use as precursors in alkali-activated systems, although their actual reactivity depends on mineralogical and processing factors [15,16,17].

To enhance the reactivity and structural performance of LWM-based binders, supplementary aluminosilicate precursors such as metakaolin (MK) and volcanic ash (VA) are commonly incorporated. MK, characterized by high amorphous silica and alumina content, and VA, often characterized by high total silica contents and variable mineralogical composition, have been widely used as supplementary precursors in alkali-activated systems due to their potential to enhance the performance of the resulting materials [18,19,20].

In parallel, EPS, a persistent plastic waste, has been explored as a lightweight aggregate in cementitious and alkali-activated composites. Its incorporation generally improves thermal insulation and reduces density; however, it often leads to reduced mechanical strength due to increased porosity and weak interfacial bonding [21,22]. Although previous studies have extensively examined EPS-modified systems based on conventional precursors such as fly ash, slag, and MK, limited attention has been given to systems derived from lignocellulosic ashes. Furthermore, most investigations have focused on bulk properties, such as density, thermal conductivity, and compressive strength, while surface-related properties, including hardness, friction behavior, and wear resistance, remain largely unexplored [23,24,25]. For lightweight alkali-activated composites intended for non-structural construction applications, surface-related properties may also be relevant because these materials are frequently subjected to handling, transportation, installation, and service conditions involving abrasion, scratching, and frictional contact. Therefore, evaluating hardness and wear behavior can provide complementary information regarding surface durability and functional performance beyond conventional bulk mechanical properties. Despite their potential relevance, tribological properties of alkali-activated composites derived from lignocellulosic ashes have received limited attention in the literature. Surface properties, such as friction and wear resistance, are important for applications involving abrasion or repeated mechanical contact. Therefore, tribological evaluation complements conventional mechanical characterization and provides a more comprehensive assessment of the potential performance of these sustainable composites.

In this context, the present study addresses these gaps by investigating alkali-activated composites produced from WA and SCBA, incorporating VA and MK as complementary reactive materials.

The role of EPS was evaluated beyond its lightweighting effect, with particular emphasis on its influence on surface-related and tribological properties. By integrating tribological evaluation into lignocellulosic ash-based alkali-activated systems, this work contributes to an area that remains relatively unexplored in the current literature.

Accordingly, the combined effects of VA, MK, and recycled EPS on the mechanical and tribological performance of WA- and SCBA-based composites were systematically evaluated. Compressive strength, Vickers microhardness, friction coefficient, and wear rate were analyzed to provide a broader understanding of the behavior of these materials for lightweight, non-structural applications. It was anticipated that the incorporation of VA and EPS would modify composite microstructure, leading to measurable changes in mechanical and tribological performance.

## 2. Materials and Methods

### 2.1. Raw Materials and Characterization

Wood ash (WA) was obtained from Quercus candicans (Indaparapeo, Michoacan, Mexico), sugarcane bagasse ash (SCBA) from Saccharum officinarum (Tacámbaro, Michoacan, Mexico), and volcanic ash (VA) was collected from El Jorullo volcano in la Huacana, Michoacan, Mexico. Commercial materials included metakaolin (MK, Alifarma, Mexico City, Mexico), expanded polystyrene (EPS, Plexa, Morelia, Michoacan, Mexico, 384.7 g/mol), and sodium hydroxide (NaOH, purity ≥ 98%, Sigma-Aldrich, St. Louis, MO, USA).

The chemical composition of the raw materials was determined by X-ray fluorescence (XRF). Since the composition of lignocellulosic ashes varies according to biomass source and combustion conditions, XRF analysis was conducted to determine the oxide composition of the specific raw materials used in this study.

Table 1 summarizes the oxide composition determined by XRF for each raw material employed in this study and loss on ignition (LOI), which represents the mass loss after heating and is generally associated with the presence of moisture, organic matter, carbonates, and other volatile constituents. WA, SCBA, and VA contained significant amounts of SiO_2_, Al_2_O_3_, and CaO, which may contribute to alkali-activation reactions depending on their mineralogical state and availability in the alkaline medium. VA (63.9 wt.% SiO_2_) and MK exhibited high total SiO_2_ and Al_2_O_3_ contents; however, the reactive fractions of these oxides were not directly quantified in the present study. WA exhibited a high CaO content (57.5 wt.%), whereas SCBA contained a considerably lower CaO content (5.77 wt.%), highlighting compositional differences that may influence the development and performance of the alkali-activated matrix [18,26].

XRF analysis provides the total oxide composition of the raw materials but does not distinguish between reactive and crystalline fractions. The actual reactivity of SiO_2_, Al_2_O_3_, and CaO depends on their mineralogical state, amorphous content, particle characteristics, and solubility under alkaline conditions. Therefore, the oxide contents reported in Table 1 should be interpreted as indicators of the chemical potential for alkali activation rather than as direct measurements of precursor reactivity.

### 2.2. Preparation of the Alkali-Activated Composites

The formulations presented in Table 2 were designed to systematically evaluate the individual and combined effects of wood ash (WA), sugarcane bagasse ash (SCBA), metakaolin (MK), volcanic ash (VA), and expanded polystyrene (EPS) on the mechanical and tribological performance of the alkali-activated composites. MK and VA were incorporated at three replacement levels (15, 35, and 65 wt.%) to investigate the influence of complementary reactive precursors over a broad compositional range. Hybrid WA–SCBA mixtures (1:1 by mass) were included to assess possible synergistic effects between the two lignocellulosic ashes, while EPS was incorporated at a constant content (2.4 wt.% of total solids) to isolate its effect on surface-related and mechanical properties.

Before mixing, all solid precursors were conditioned to ensure consistency. Wood ash (WA) and sugarcane bagasse ash (SCBA) were sieved to a particle size range of 250–420 µm, metakaolin (MK) to <25 µm, volcanic ash (VA) to <18 µm, and EPS particles to 1.5–2.0 mm. All materials were oven-dried at 105 °C for 24 h and stored in sealed containers to avoid moisture uptake before use.

The alkaline activating solution was prepared by dissolving sodium hydroxide pellets in distilled water to obtain a 15 M solution. The solution was prepared at least 24 h in advance, allowed to cool to room temperature (25 ± 2 °C), and stored until use to ensure thermal equilibrium and complete dissolution [27]. For each formulation (Table 2), the solid precursors (WA, SCBA, or WA–SCBA blends combined with MK or VA) were first dry-mixed in a mechanical mixer for 3 min to ensure homogeneity. Subsequently, the 15 M NaOH solution was gradually added while mixing continued for an additional 5 min at medium speed [28]. The liquid-to-solid (L/S) ratio was maintained at 0.65 for all formulations to ensure consistent workability and adequate flowability. For mixtures incorporating EPS, 2.4 wt.% EPS (relative to total solids) was added after the initial wet mixing stage and manually blended for 2 min to ensure uniform dispersion without excessive particle breakage. The fresh alkali-activated pastes were immediately poured into stainless steel cubic molds (25 × 25 × 25 mm^3^) in two layers. Each layer was compacted using mechanical vibration for 20 s to eliminate entrapped air and improve packing density. The molds were then covered with polyethylene film to minimize moisture loss and prevent surface cracking. The initial curing was carried out in a controlled humidity chamber at 99 ± 1% relative humidity and 25 ± 2 °C for 24 h. After demolding, specimens were subjected to a stepwise thermal curing regime: 2 h at 60 °C, followed by 2 h at 90 °C, and finally 7 h at 110 °C. This thermal treatment was applied to promote alkali-activation reactions and enhance mechanical performance. After curing, samples were allowed to cool gradually to room temperature inside the switched-off oven and stored under controlled laboratory conditions (25 ± 2 °C and ~50% RH) until further testing [29].

### 2.3. Characterization of the Alkali-Activated Composites

Compressive strength was evaluated using a universal testing machine (Bruf, Germany) at a loading rate of 0.98 kN/min. Five specimens were tested for each formulation after 28 days of curing, and the reported values correspond to the mean ± standard deviation (*n* = 5). The testing procedure was adapted from ASTM C109/C109M-16 [30] because smaller cubic specimens were used.

Vickers microhardness (HV0.1) was measured using a Mitutoyo microhardness tester HM-124 (Mitutoyo Corporation, Kawasaki, Japan) in accordance with UNE 7-423-84 [31]. Ten indentations were performed on each specimen using a load of 100 gf (0.98 N), a dwell time of 15 s, and a 40× objective lens. Indentations were placed in representative surface regions, avoiding visible pores, cracks, and large inclusions. Reported hardness values correspond to the mean ± standard deviation of the indentations (*n* = 10).

Due to the heterogeneous nature of the alkali-activated composites, the obtained values should be interpreted as localized surface microhardness measurements that reflect resistance to indentation rather than the bulk mechanical behavior of the material.

Statistical analyses were performed using one-way analysis of variance (ANOVA) to evaluate the effect of formulation composition and EPS incorporation on the measured properties. When significant differences were detected, Tukey’s honestly significant difference (HSD) post hoc test was applied for pairwise comparisons. Statistical significance was established at a confidence level of 95% (α = 0.05). All results are reported as mean ± standard deviation.

Tribological properties were evaluated following DIN 50324 and ASTM G99-17 standards [32,33], focusing on the coefficient of friction and wear rate of the composites. Tests were carried out using a ball-on-Disc tribometer under a normal load of 5 N using a 6 mm diameter alumina ball, at a constant sliding speed of 0.01 m/s, with a track radius of 5 mm and a total sliding distance of 5 m. All experiments were conducted in triplicate under dry conditions and at room temperature. Tribological testing was performed on formulations 3A, 6A, 9A, 3B, 6B, and 9B and their EPS-containing counterparts. These formulations were selected because they contained the highest proportion of the complementary precursor within each WA-, SCBA-, and hybrid WA–SCBA-based family, enabling a consistent comparison between MK- and VA-containing systems.

## 3. Results and Discussion

### 3.1. Visual Analysis of Composites

**Figure 1:**
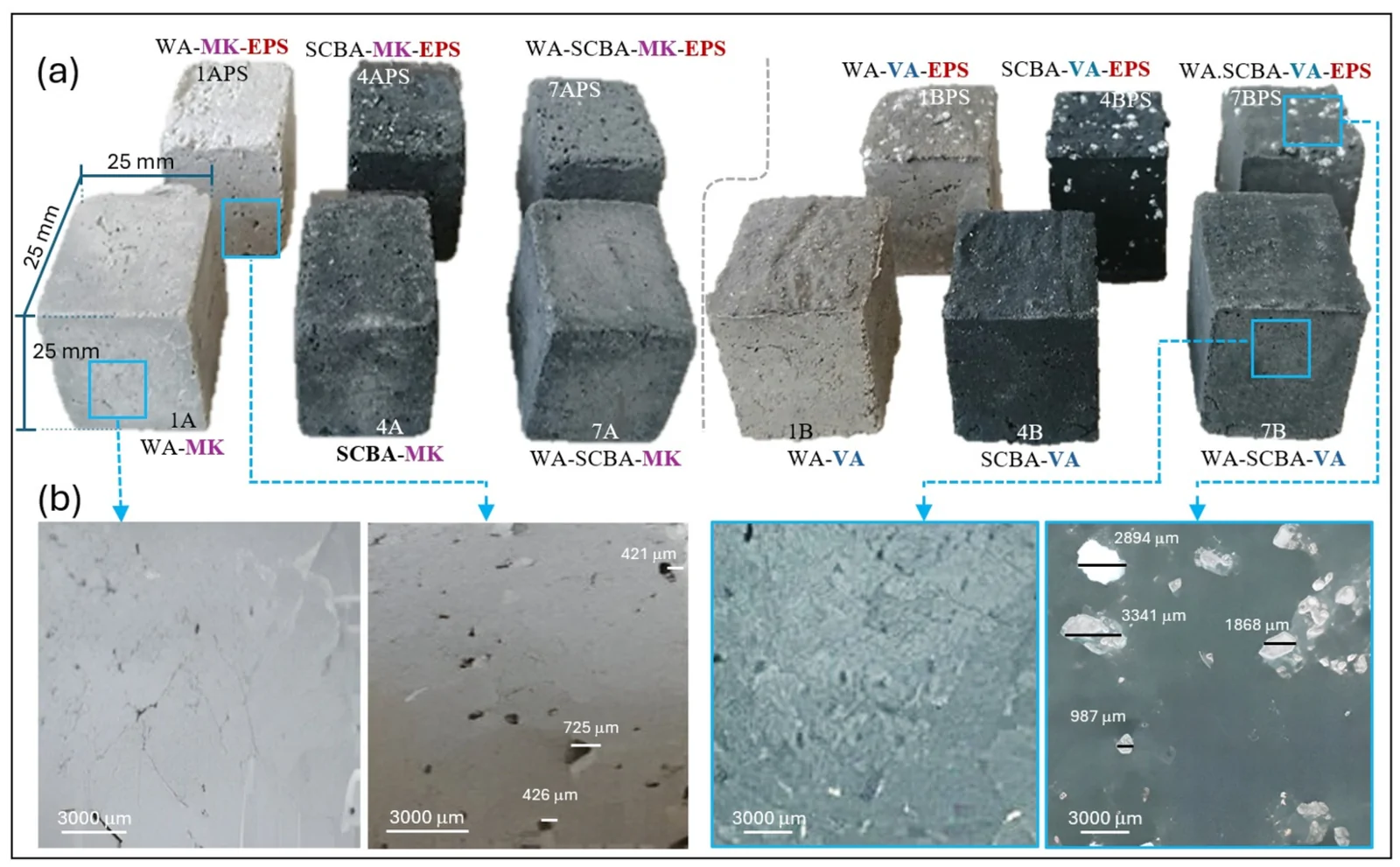
(**a**) Representative digital images of composites with WA and SCBA combined with MK or VA, with and without EPS. (**b**) Representative optical microscopy images (40×) of the selected regions indicated in (**a**).

Figure 1 presents representative macroscopic and optical microscopy images of the alkali-activated composites. The observed surface morphology provides qualitative context for interpreting the compressive strength, hardness, and tribological behavior discussed in the following sections.

VA-containing composites (WA–VA, SCBA–VA, and WA–SCBA–VA) generally exhibited smoother and more homogeneous surfaces than the corresponding MK-based formulations (WA–MK, SCBA–MK, WA–SCBA–MK). This behavior may be related to the finer particle size of VA, which can promote improved particle packing and a more homogeneous matrix. Similar visual characteristics have been reported for alkali-activated materials containing VA [34]. In contrast, EPS–containing composites displayed visible polymeric inclusions and localized voids, resulting in a more heterogeneous morphology. These features are consistent with the reduction in compressive strength observed after EPS incorporation and support the proposed disruption of matrix continuity. Furthermore, in some VA-containing composites, EPS particles appeared visually more uniformly distributed throughout the observed surface area than in the corresponding MK-based systems.

Although qualitative, these observations provide complementary evidence that helps interpret the mechanical and tribological behavior of the composites. The smoother and more homogeneous surfaces observed in the VA-containing formulations are consistent with improved particle packing and matrix continuity, which may explain their generally higher compressive strength, hardness, and wear resistance. Conversely, the localized voids and polymeric inclusions observed in the EPS-containing composites are consistent with the reduction in compressive strength and indicate interruptions in matrix continuity. Although optical microscopy does not permit direct identification of reaction products or pore structure, it provides useful qualitative evidence linking surface morphology with the measured mechanical and tribological properties. Further microstructural characterization using higher-resolution techniques, such as scanning electron microscopy (SEM), coupled with elemental or phase analysis, is required to establish direct structure–property relationships.

### 3.2. Compressive Strength

**Figure 2:**
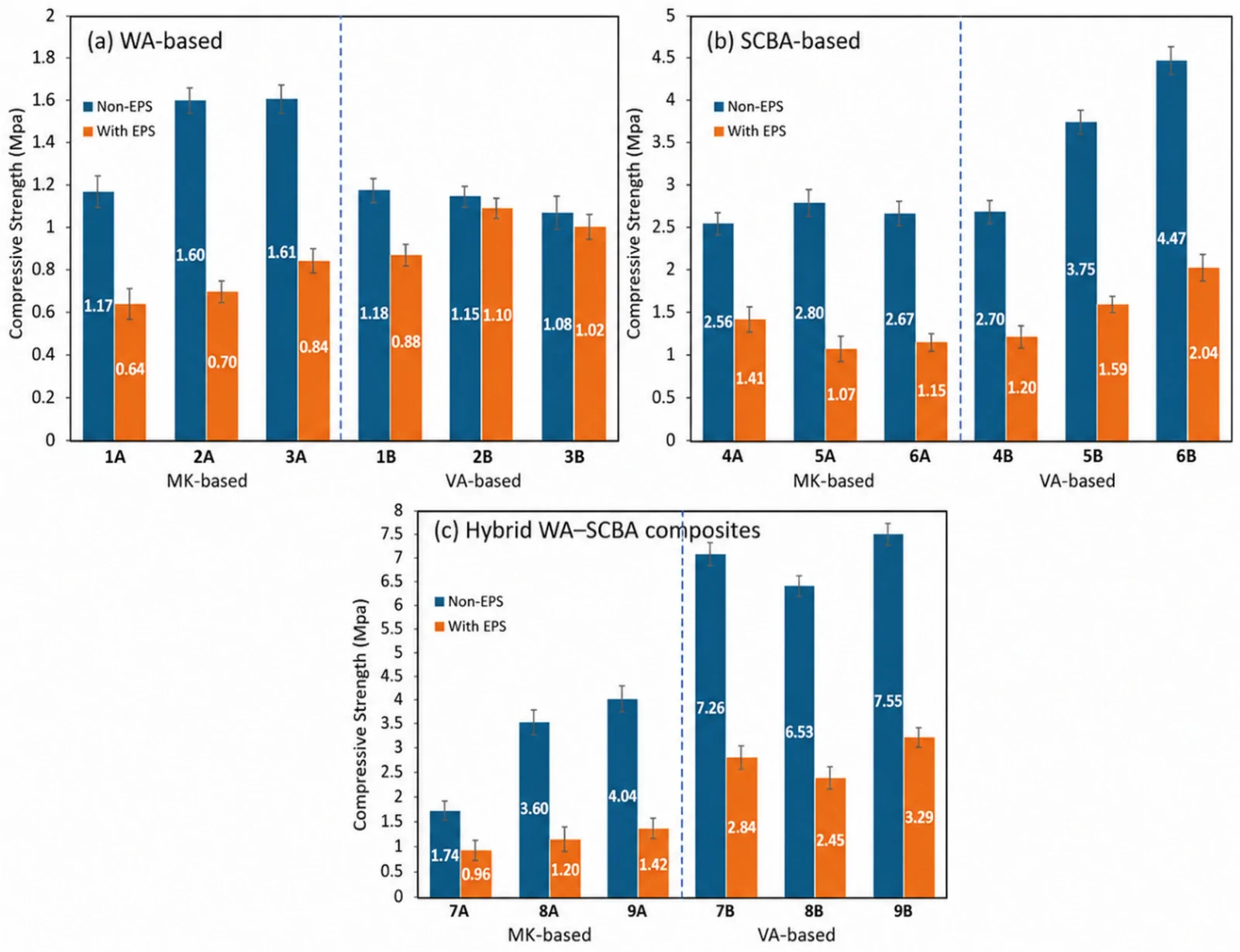
Effect of expanded polystyrene (EPS) incorporation on the compressive strength of alkali-activated composites: (**a**) WA-based, (**b**) SCBA-based, and (**c**) hybrid WA–SCBA composites. In each subfigure, MK- and VA-based formulations are separated by the vertical dashed line. Values represent mean ± standard deviation (*n* = 5). Each subfigure uses an independent *y*-axis scale.

The compressive strength results (Figure 2) are discussed in the context of the morphological features previously observed in the composites, which have been reorganized to display each composition individually and further analyzed according to precursor type (WA, SCBA, and hybrid WA–SCBA systems) and matrix formulation (MK- and VA-based). Reported values correspond to the mean ± standard deviation obtained from five specimens (*n* = 5).

Overall, the results indicate that compressive strength is governed by a complex interplay between precursor chemistry, calcium availability, particle size distribution, and matrix densification, rather than by the individual content of SiO_2_ and Al_2_O_3_ alone. Additionally, the incorporation of EPS consistently reduced compressive strength across all systems due to increased porosity and weak interfacial bonding.

#### 3.2.1. WA-Based Systems

In WA-based composites (Figure 2a), MK-based formulations (1A–3A) exhibited slightly higher compressive strength (up to ~1.61 MPa) compared to VA-based systems (1B–3B), which reached values of ~1.18 MPa. This behavior could be attributed to differences in precursor composition, particle size, and calcium/silica availability. Although WA contains a high CaO content (57.50%), its mechanical contribution may be limited by its heterogeneous chemical and physical characteristics; however, the amorphous fraction and intrinsic reactivity were not directly quantified. In VA-based systems, the finer particle size (≤18 µm) improves packing density; however, this packing effect alone did not produce higher compressive strength than the corresponding MK-based formulations.

The addition of EPS led to a significant reduction in compressive strength (approximately 30–50%), confirming that the introduction of polymeric inclusions disrupts matrix continuity and reduces load-bearing capacity.

#### 3.2.2. SCBA-Based Systems

A markedly different trend was observed in SCBA–based systems (Figure 2b), where VA-based formulations, particularly 6B, achieved the highest compressive strength within this group (~4.47 MPa). This enhanced performance is primarily attributed to the high silica content of SCBA (57.50%), which can increase the reactivity of the precursor mixture under alkaline conditions and contribute to improved matrix cohesion [35,36]. In addition, the fine particle size of VA may enhance particle packing and promote a more homogeneous distribution of the solid phases within the matrix [37].

Despite the high aluminosilicate content of MK, the lower compressive strengths obtained in MK-containing formulations indicate that mechanical performance was influenced by multiple factors beyond chemical composition alone. It is worth noting that MK was incorporated as a complementary component together with SCBA rather than evaluated as a standalone precursor, the resulting behavior reflects the combined effect of precursor interactions, particle characteristics, and processing conditions. Overall, the results suggest that the combination of SCBA and VA favored the development of a more cohesive alkali-activated matrix, resulting in higher compressive strength than the corresponding MK-containing systems.

#### 3.2.3. Hybrid WA–SCBA Systems

Hybrid WA–SCBA systems (Figure 2c) exhibited the highest compressive strength across all formulations, particularly in VA–based composites. Formulation 9B achieved the maximum value (7.55 ± 0.25 MPa), surpassing all WA-based (1.08–1.61 MPa) and SCBA–based formulations (maximum 4.47 MPa). This enhanced performance is likely associated with the synergistic interaction between the calcium-rich WA (57.5 wt.% CaO) and the silica-rich SCBA and VA (57.5 and 63.9 wt.% SiO_2_, respectively). The high silica content and fine particle size of VA (<18 μm) may have enhanced precursor reactivity and particle packing, favoring the development of a denser alkali-activated matrix. This balanced CaO/SiO_2_ composition may favor the development of a more cohesive alkali-activated matrix, which could contribute to the observed strength enhancement [28,37,38,39].

In contrast, the corresponding MK-containing formulation (9A) exhibited a lower compressive strength (~4.04 MPa), indicating that the performance of these hybrid systems depends not only on the presence of reactive aluminosilicate phases but also on the interactions among the different precursors and the resulting matrix structure. It is important to emphasize that MK was evaluated as a complementary precursor together with WA and SCBA rather than as a sole aluminosilicate source; therefore, the measured strength values reflect the behavior of the hybrid formulations rather than the intrinsic performance of alkali-activated metakaolin systems.

#### 3.2.4. Comparative Analysis Across Precursor Types and Matrices

When comparing all systems, it is evident that no single precursor or matrix type universally dominates performance. Instead, compressive strength is controlled by the synergy between chemical composition, particle size distribution, packing density, and the extent of alkali-activation reactions.

MK-based systems tend to provide stable but moderate strength due to their high aluminosilicate reactivity. Since MK was evaluated as a complementary mineral additive in combination with WA, SCBA, or WA–SCBA blends, the mechanical response reflects the behavior of the hybrid formulations rather than that of alkali-activated metakaolin alone. In contrast, VA-based systems, although less reactive chemically, can achieve superior performance when combined with appropriate precursors, particularly when calcium is available from sources such as WA. The highest strengths are consistently observed in hybrid systems, confirming that combining precursors with complementary chemical compositions is an effective strategy to enhance mechanical performance.

Overall, the mechanical performance of the composites appears to be governed by the balance between precursor oxide composition and reactivity rather than by the concentration of any individual component. This observation highlights the importance of considering the combined effects of SiO_2_, Al_2_O_3_, CaO, and LOI when designing alkali-activated systems derived from heterogeneous waste materials.

#### 3.2.5. Effect of EPS Incorporation

Across all formulations, EPS incorporation resulted in a reduction in compressive strength ranging from approximately 30% to 60%. This decrease is attributed to increased porosity within the matrix, weak interfacial adhesion between EPS and the inorganic binder, and reduced effective load-bearing cross-section [38]. Despite this reduction in compressive strength, hybrid systems, particularly the VA-based formulations, exhibited the highest strength values among the EPS-containing composites. Although their mechanical performance is insufficient for structural applications, the obtained strength levels suggest potential use in non-load-bearing building materials, such as lightweight partition panels, interior wall components, and other applications where reduced weight and moderate mechanical resistance are required.

### 3.3. Vickers Hardness of the Composites

Vickers microhardness evaluates localized resistance to surface indentation and should not be directly correlated with bulk mechanical behavior, particularly in heterogeneous alkali-activated composites containing pores, unreacted particles, and EPS inclusions.

All composites incorporating EPS exhibited higher Vickers hardness values than their corresponding formulations without EPS (Table 3). Average hardness increased from 11.3–15.2 HV to 16.2–23.1 HV after EPS incorporation. Among the formulation groups, WA–SCBA–VA composites exhibited the highest average hardness (23.1 HV), whereas the highest individual value was recorded for formulation 9BPS (25.4 HV).

Although EPS incorporation reduced compressive strength, it increased Vickers hardness in several formulations. This apparent contrast can be explained by the different deformation mechanisms involved in each test. Compressive strength reflects the load-bearing capacity of the entire composite and is strongly influenced by matrix continuity, interfacial bonding, and internal porosity. In contrast, Vickers hardness measures localized resistance to plastic deformation within a small surface area. The increase in surface hardness cannot be attributed to a specific EPS-related mechanism based on the available characterization. However, the apparent contrast with compressive strength can be understood because the two tests probe different length scales: compressive strength reflects the global load-bearing capacity, whereas Vickers hardness measures localized resistance to indentation. Therefore, an increase in local surface resistance may coexist with a reduction in bulk strength caused by pores and interfacial discontinuities. Similar behavior has been reported for lightweight geopolymer and EPS-containing composites, where local surface properties and bulk mechanical performance are governed by different mechanisms [23,24,38,40,41].

Among the formulations evaluated, composites containing VA generally exhibited slightly higher hardness values than the corresponding MK-containing systems. This trend was particularly evident in the WA–SCBA formulations. Although differences in precursor composition and particle size may contribute to the observed behavior [39,41], the consistently higher hardness values observed in VA-containing systems may be related to their finer particle size (<18 µm), which may favor a more uniform surface texture and greater resistance to localized indentation.

The hardness increase observed after EPS incorporation may be associated with localized surface changes induced by EPS addition and thermal curing (up to 110 °C), which could have modified the morphology of the EPS inclusions and, consequently, the local indentation response.

However, the mechanisms responsible for this behavior cannot be directly established based on the present results. Consequently, the higher hardness values should be interpreted as an increase in localized surface resistance rather than as evidence of improved bulk mechanical performance.

### 3.4. Friction Coefficient and Wear Rate of Composites

The tribological results indicate that the composition of the alkali-activated composites influenced both the coefficient of friction (COF) and wear rate. In general, differences were observed among formulations containing WA, SCBA, MK, VA, and EPS, demonstrating that precursor selection affected the surface response under sliding conditions.

**Figure 3:**
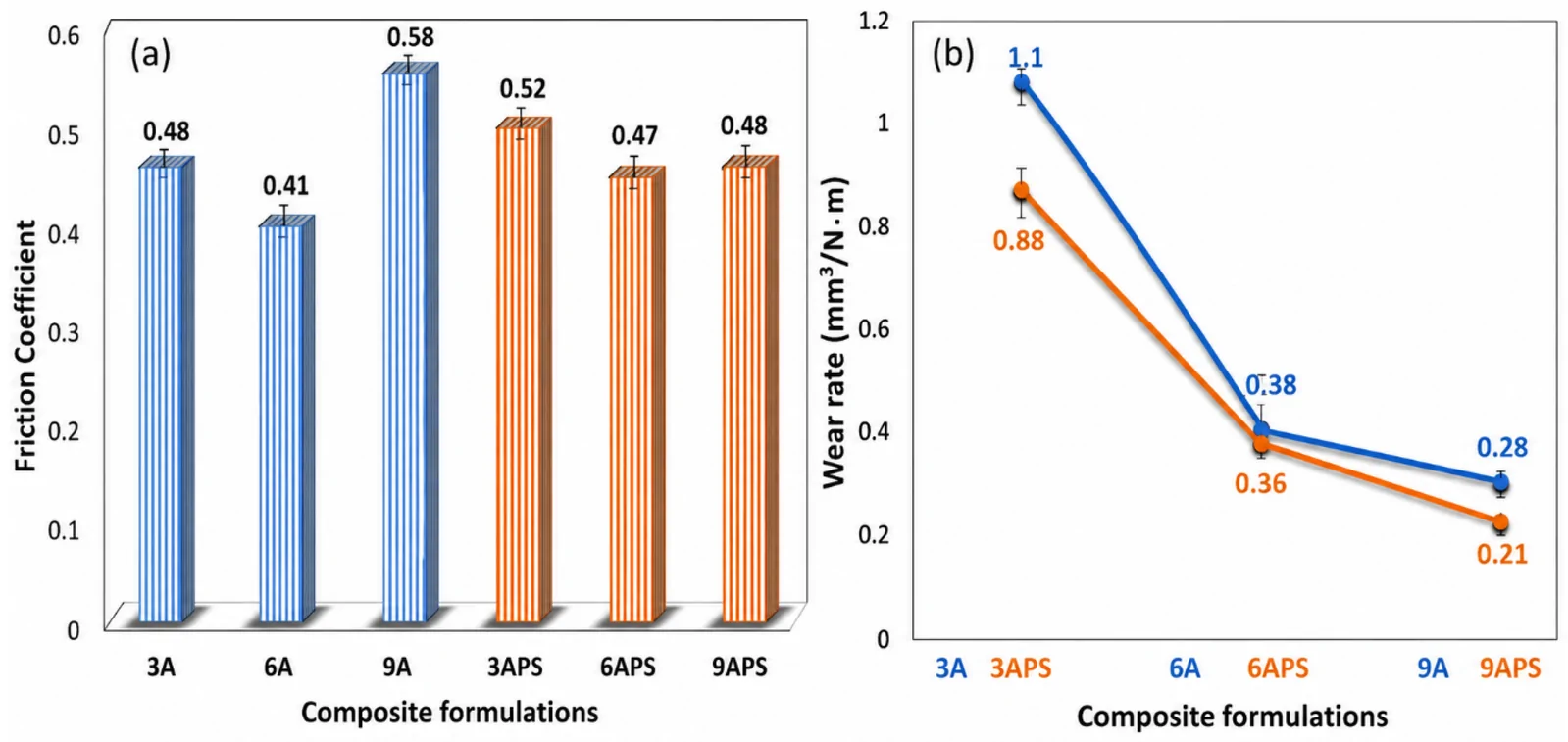
Tribological behavior of MK-based composites with and without EPS: (**a**) coefficient of friction (COF) and (**b**) wear rate. Values represent mean ± standard deviation (*n* = 3). Error bars indicate standard deviation. Blue and orange represent composites without and with EPS, respectively.

**Figure 4:**
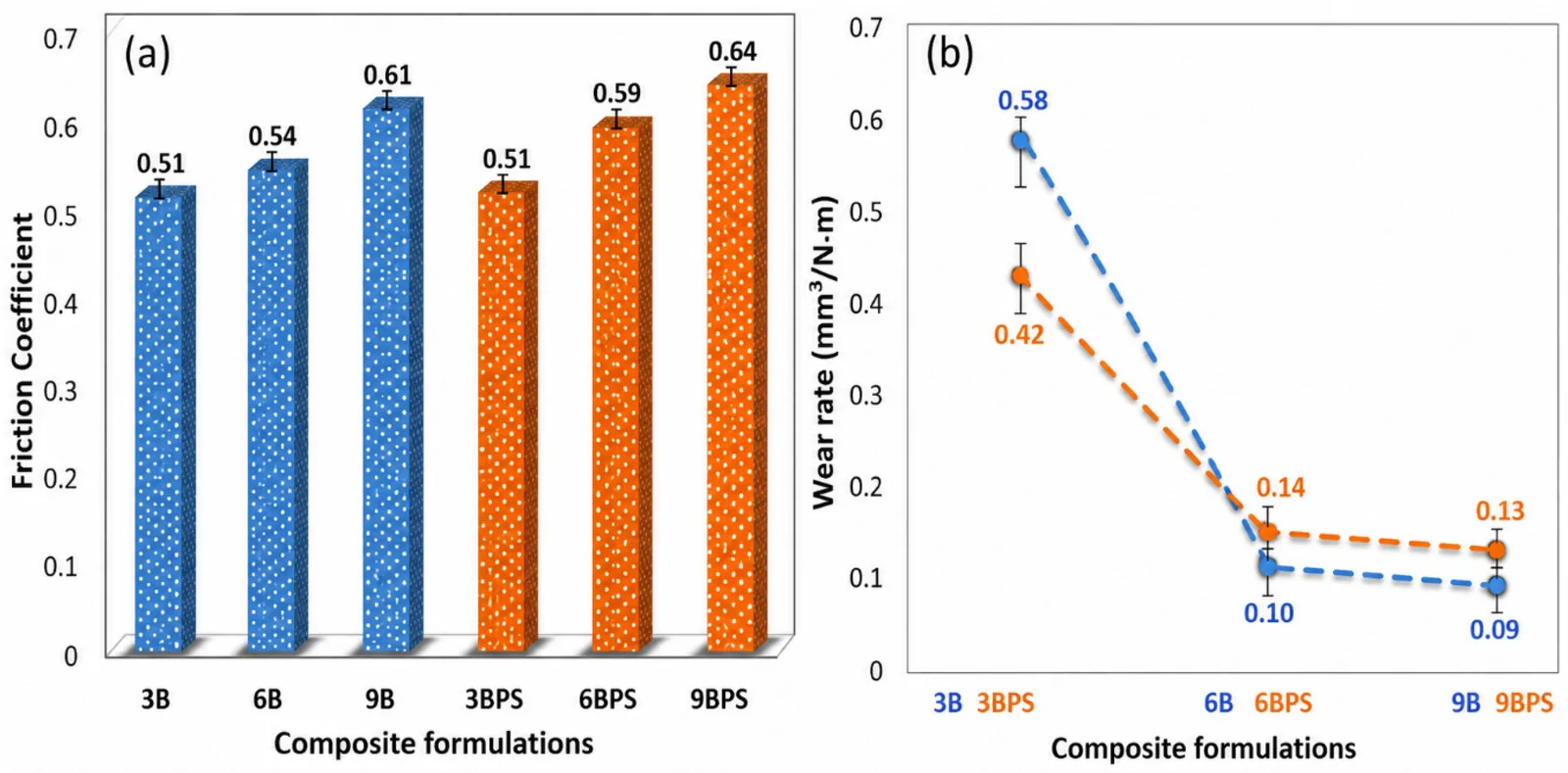
Tribological behavior of VA-based composites with and without EPS: (**a**) coefficient of friction (COF) and (**b**) wear rate. Values represent mean ± standard deviation (*n* = 3). Error bars indicate standard deviation. Blue and orange represent composites without and with EPS, respectively.

For the MK-based systems (Figure 3), the COF increased from 0.41 for the SCBA–MK formulation (6A) to 0.58 for the WA–SCBA–MK formulation (9A), while the wear rate decreased from 0.38 to 0.28 mm^3^/N·m. In VA-based systems, the WA–SCBA–VA formulation (9B) (Figure 4), exhibited the highest COF (0.61) but the lowest wear rate (0.09 mm^3^/N·m). Previous studies have reported microstructural changes in metakaolin-based geopolymers containing volcanic ash [42]. In the present study, the lower wear rate of the VA-containing hybrid formulation coincided with a smoother observed surface and higher hardness; however, a direct microstructural mechanism cannot be established without wear-track and phase characterization.

These observations indicate also that COF and wear rate were not directly correlated and suggest that the incorporation of WA influenced the tribological response of SCBA-based systems. Similar behavior has been reported in heterogeneous mineral-based composites [43,44]. The lower wear rates observed for SCBA-rich and WA–SCBA–VA composites may be associated with their higher SiO_2_ content of SCBA (57.5 wt.%) and VA (63.9 wt.%), together with the fine particle size of VA (<18 μm), which could contribute to improved surface resistance during sliding [44].

An inverse trend between Vickers hardness and wear rate was observed. Formulations 9B and 9BPS exhibited the highest hardness values (15.6 and 25.4 HV respectively), together with the lowest wear rates (0.09 and 0.13 mm^3^/N·m respectively), suggesting a possible association between increased surface hardness and reduced material loss during sliding. Consequently, higher COF values do not automatically imply lower wear resistance in these heterogeneous composite systems. Nevertheless, additional characterization of wear tracks and wear debris would be required to determine whether this behavior is associated with abrasive, adhesive, fatigue, or mixed wear mechanisms.

It should be noted that the composites were subjected to thermal curing treatment during fabrication. Under these conditions, partial softening or morphological modification of the EPS particles may have occurred [18,42,45]. However, no specific characterization of the EPS phase after curing was performed in the present study. Therefore, the influence of possible EPS deformation on the observed tribological behavior cannot be established and remains a topic for future investigation.

Although wear-track characterization was not performed in the present study, the relatively low wear rates observed for several formulations indicate differences in their resistance to material removal under sliding conditions. However, the dominant wear mechanisms cannot be identified from the available results. Therefore, the possible occurrence of abrasive, adhesive, fatigue, or mixed wear mechanisms, as well as the formation of compacted debris layers or tribofilms, requires confirmation through direct examination of wear tracks and wear debris morphology.

Direct comparison of tribological properties reported for alkali-activated materials remains challenging because wear behavior is strongly influenced by testing conditions, including normal load, sliding speed, counterface material, and sliding distance. Nevertheless, previous studies generally associate greater surface hardness and matrix integrity with reduced material removal during sliding. The present results are consistent with this trend, as the WA–SCBA–VA formulation exhibited the lowest wear rate (0.09 mm^3^/N·m), which coincided with improved mechanical performance and a more homogeneous surface morphology.

Similarly, Sydow et al. [44] reported that composites reinforced with agricultural and industrial residues commonly exhibit improved tribological performance when reinforcement promotes higher hardness and better load distribution. Likewise, Barve and Khairnar [43] demonstrated that sugarcane bagasse ash can effectively enhance friction material performance by increasing wear resistance, supporting the beneficial role of lignocellulosic ash observed in the present investigation. Although direct quantitative comparisons remain limited because of differences in material composition and test conditions, the overall trends are in good agreement with the available literature. The scarcity of tribological studies on lignocellulosic ash-based alkali-activated composites highlights the novelty of the present work, which provides one of the first systematic comparisons of wear behavior among WA-, SCBA-, and hybrid WA–SCBA-based systems modified with MK, VA, and recycled EPS.

Overall, the results indicate that surface performance was strongly governed by precursor composition. The combination of WA, SCBA, and VA provided a more favorable balance between matrix integrity, hardness, and wear resistance than the corresponding MK-based formulations. The higher silica content and finer particle size of VA may contribute to a denser and more homogeneous alkali-activated matrix, whereas EPS primarily modified local deformation behavior by increasing surface hardness while reducing bulk compressive strength due to matrix discontinuities. These findings demonstrate that precursor composition plays a key role in controlling the mechanical and tribological response of lightweight alkali-activated composites.

## 4. Conclusions

The mechanical and tribological properties of the alkali-activated composites were strongly influenced by precursor composition.

Among all formulations, the WA–SCBA–VA system (9B) exhibited the highest compressive strength (7.55 ± 0.25 MPa), which may be associated with the combined contribution of calcium-rich WA, silica-rich SCBA and VA, and the fine particle size of VA, favoring improved particle packing under the investigated conditions.

EPS reduced compressive strength but increased surface hardness. The highest Vickers hardness value was obtained for formulation 9BPS (25.4 HV), suggesting that EPS incorporation and thermal curing may have influenced the localized surface response; however, the underlying mechanism was not directly characterized. Because hardness reflects resistance to localized indentation rather than bulk mechanical behavior, these results should be interpreted as indicators of surface performance rather than structural capacity.

Tribological performance was strongly affected by precursor composition and EPS addition, demonstrating that precursor composition was an important factor governing surface performance under the investigated conditions. The WA–SCBA–VA formulation (9B) exhibited the lowest wear rate (0.09 mm^3^/N·m), while composites with higher hardness generally tended to show lower wear rates, suggesting that increased resistance to localized surface deformation contributed to improved wear resistance under the applied sliding conditions. The combined mechanical and tribological results indicate that under the investigated conditions, volcanic ash generally resulted in better overall performance than metakaolin when incorporated into WA–SCBA systems. The superior performance of VA-containing composites may be associated with their high silica content and fine particle size, which likely improved particle packing and matrix consolidation, thereby enhancing hardness and wear resistance.

Overall, this study demonstrates that lignocellulosic ashes, volcanic ash, and recycled EPS can be successfully valorized to produce lightweight alkali-activated composites with tunable mechanical and tribological performance. Furthermore, it provides one of the few comparative assessments of friction and wear behavior in lignocellulosic ash-based alkali-activated composites. This assessment complements conventional mechanical characterization and provides new insight into the potential applications of these materials in components subjected to surface contact and wear.

Because the oxide composition of lignocellulosic ashes depends on biomass source and combustion conditions, the quantitative results reported in this study are specific to the raw materials characterized by XRF. Variations in SiO_2_, CaO, Al_2_O_3_, and LOI are expected to influence alkali-activation reactions and, consequently, the mechanical and tribological performance of the resulting composites.

Future research should focus on wear-track characterization by SEM, wear-debris analysis, and phase identification to establish direct structure–property relationships and clarify the mechanisms governing friction and wear in these materials.

These results demonstrate that lignocellulosic ashes can be an alternative precursor for lightweight alkali-activated composites, contributing to waste utilization and circular economy strategies.

## Figures and Tables

**Table 1:** Oxide compositions (wt.%) and LOI (%) of starting materials determined by XRF (wt.%).

Material	SiO_2_	Al_2_O_3_	K_2_O	CaO	Fe_2_O_3_	Loss on Ignition (LOI)%
WA	3.98	1.51	9.89	57.50	1.19	8.50
SCBA	57.50	6.30	11.00	5.77	4.16	9.80
VA	63.90	10.40	5.80	4.70	6.20	6.31
MK	53.00	41.40	0.26	0.31	0.77	1.16

**Table 2:** Experimental formulation matrix used to evaluate the effects of precursor composition and EPS incorporation on alkali-activated composites.

Formulations	WA	SCBA	VA	MK	Non-EPS	Formulations	With EPS *
WA-MK	85	-	-	15	1A	WA-MK-EPS	1APS
WA-MK	65	-	-	35	2A	WA-MK-EPS	2APS
WA-MK	35	-	-	65	3A	WA-MK-EPS	3APS
WA-VA	85	-	15	-	1B	WA-VA-EPS	1BPS
WA-VA	65	-	35	-	2B	WA-VA-EPS	2BPS
WA-VA	35	-	65	-	3B	WA-VA-EPS	3BPS
SCBA-MK	-	85	-	15	4A	SCBA-MK-EPS	4APS
SCBA-MK	-	65	-	35	5A	SCBA-MK-EPS	5APS
SCBA-MK	-	35	-	65	6A	SCBA-MK-EPS	6APS
SCBA-VA	-	85	15	-	4B	SCBA-VA-EPS	4BPS
SCBA-VA	-	65	35	-	5B	SCBA-VA-EPS	5BPS
SCBA-VA	-	35	65	-	6B	SCBA-VA-EPS	6BPS
WA-SCBA-MK	42.5	42.5	-	15	7A	WA-SCBA-MK-EPS	7APS
WA-SCBA-MK	32.5	32.5	-	35	8A	WA-SCBA-MK-EPS	8APS
WA-SCBA-MK	17.5	17.5	-	65	9A	WA-SCBA-MK-EPS	9APS
WA-SCBA-VA	42.5	42.5	15	-	7B	WA-SCBA-VA-EPS	7BPS
WA-SCBA-VA	32.5	32.5	35	-	8B	WA-SCBA-VA-EPS	8BPS
WA-SCBA-VA	17.5	17.5	65	-	9B	WA-SCBA-VA-EPS	9BPS

* Code after adding 2.4 wt.% EPS into all WA- and SCBA-based formulations.

**Table 3:** Effect of EPS on the Vickers hardness of WA- and SCBA-based composites containing MK and VA.

Formulation	Non-EPS	HV (*n* = 10)	SD	Mean HV	With EPS	HV (*n* = 10)	SD	Mean HV
WA-MK	1A	14.0	2.5		1APS	16.4	3.2	
WA-MK	2A	13.0	3.1	14.0	2APS	16.8	2.4	16.6
WA-MK	3A	15.0	2.9		3APS	16.5	3.2	
WA-VA	1B	15.1	1.7		1BPS	17.1	1.3	
WA-VA	2B	13.1	0.9	14.4	2BPS	16.8	3.7	16.8
WA-VA	3B	15.1	2.5		3BPS	16.4	3.0	
SCBA-MK	4A	14.5	2.3		4APS	16.3	2.5	
SCBA-MK	5A	14.7	2.1	14.7	5APS	18.8	3.0	17.5
SCBA-MK	6A	14.9	2.4		6APS	17.5	2.6	
SCBA-VA	4B	13.9	2.0		4BPS	17.5	1.8	
SCBA-VA	5B	13.8	0.7	14.7	5BPS	18.2	2.5	17.6
SCBA-VA	6B	16.5	2.3		6BPS	17.0	0.9	
WA-SCBA-MK	7A	9.5	2.2		7APS	17.0	3.2	
WA-SCBA-MK	8A	10.5	2.5	11.3	8APS	15.5	3.0	16.2
WA-SCBA-MK	9A	14.0	2.0		9APS	16.2	2.8	
WA-SCBA-VA	7B	14.6	2.2		7BPS	22.5	2.7	
WA-SCBA-VA	8B	15.3	2.5	15.2	8BPS	21.5	2.8	23.1
WA-SCBA-VA	9B	15.6	2.7		9BPS	25.4	0.8	

Values correspond to the mean ± standard deviation obtained from ten indentations (*n* = 10).

## Data Availability

The original contributions presented in this study are included in the article. Further inquiries can be directed to the corresponding authors.

## Abbreviations

The following abbreviations are used in this manuscript:

AAM	Alkali-Activated Material
ANOVA	Analysis of Variance
COF	Coefficient of Friction
EPS	Expanded Polystyrene
HSD	Honestly Significant Difference
HV	Vickers Hardness
LWM	Lignocellulosic Waste Materials
MK	Metakaolin
SCBA	Sugarcane Bagasse Ash
VA	Volcanic Ash
WA	Wood Ash
XRF	X-ray Fluorescence
